# Upper body and lower limbs musculoskeletal symptoms and health inequalities in Europe: an analysis of cross-sectional data

**DOI:** 10.1186/1471-2474-15-285

**Published:** 2014-08-26

**Authors:** Diego Montano

**Affiliations:** Faculty of Medicine, Senior professorship “Work Stress Research”, Duesseldorf University, Merowingerplatz. 1a, 40225 Duesseldorf, Germany

**Keywords:** Social determinants of health, Musculoskeletal symptoms in Europe, Occupational diseases, Lower limbs symptoms, Upper limbs and/or shoulder and neck symptoms

## Abstract

**Background:**

Musculoskeletal disorders are the most frequent occupational diseases in Europe. However, their multifactorial aetiology poses several challenges concerning not only the estimation of relative prevalence rates across occupational groups but also how the co-occurrence of known risk factors might differ between disorders of the upper and lower limbs. Against this background, the following objectives are pursued: (1) to estimate the relative odds and prevalence rates of self-reported disorders of the upper limbs and/or shoulders and neck (upper body) and the lower limbs for major ISCO-88 occupational groups, (2) to evaluate to what extent the associations between known risk factors differ for musculoskeletal disorders of the upper body and the lower limbs.

**Methods:**

Statistical analysis of cross-sectional data from the European Working Conditions Survey 1995-2010. The probability of reporting upper body and lower limbs pain in the survey sample 2010 is estimated by mixed logistic regression models using the Markov chain Monte Carlo Sampler. Independent variables include some known physical and psychosocial risk factors.

**Results:**

Concerning the first objective, an excess risk of reporting musculoskeketal disorders of the upper body was observed among craft workers (ISCO 7), machine operators (ISCO 8) and workers in elementary occupations (ISCO 9). Concerning musculoskeletal disorders of the lower limbs, service and sales workers (ISCO 5) and workers in ISCO groups 7, 8 and 9 reported symptoms more frequently. Regarding the second objective, similar association patterns were observed for upper body and lower limbs symptoms. Major physical risk factors associated with both symptom types were very frequent exposure to tiring positions, carrying heavy loads and performing repetitive tasks. Standing appears to be an important risk factor for lower limbs symptoms only.

**Conclusions:**

Results suggest that the unequal burden of exposure has not changed substantially across occupational groups since 1995, and that there is urgent need of delivering and evaluating the effects of specific interventions targeting workers at high risk of developing musculoskeletal disorders.

**Electronic supplementary material:**

The online version of this article (doi:10.1186/1471-2474-15-285) contains supplementary material, which is available to authorized users.

## Background

Musculoskeletal disorders (MSD, ICD-10 codes M00-M99) are the second most frequent medical cause underlying disability benefit claims in OECD countries [1], and the most frequent occupational disease in Europe (EU-15) [2]. Although prevalence rates of MSD vary widely depending on the body regions considered and the instruments utilised for the assessment of symptoms, prevalence rates of more than 30% have been reported in several European epidemiological studies [3–5]. Extensive review articles have identified major work-related physical risk factors of MSD such as repetitive movements, high-force demands, awkward or extreme positions, rapid work pace, extreme temperatures, insufficient recovery time, mechanical pressure concentrations, and segmental or whole-body vibration [6–8]. Psychosocial risk factors, especially high demands and low control at the workplace [9], have been identified as additional risk factors [10]. Main aetiologic mechanisms of MSD and physical and psychosocial risk factors seem to be based mainly on the relationships between biomechanical load and corresponding pathophysiologic alterations of tissues, and stress-induced alterations of the neurohormonal system, pain perception, hippocampal neurogenesis and gene expression, respectively [6, 11].

Nevertheless, work-related risk factors are unequally distributed across occupational groups depending on the specific nature of the work tasks and production processes, ergonomic characteristics of the workplace, psychosocial characteristics of the work environment and national and organisational occupational health policies. For instance, prospective epidemiological evidence from a twin study in Sweden reported statistically significant excess risk of obtaining disability pension due to MSD among workers employed in the health care and social sector, transport, production and mining, services and military [12]. Concerning physical risk factors, another prospective twin study in Finland confirmed that shift work, monotonous work, lifting and carrying, physically demanding work are strongly associated with work incapacity and disability pension [13]. The results of both studies were consistent after controlling for genetic and shared-environment factors. Prevalence rates of self-reported MSD by occupational ISCO groups from European cross-sectional data are highest among service and sales workers (ISCO 5), elementary occupations (ISCO 9), plant and machine operators (ISCO 8), and skilled agricultural and fishery workers (ISCO 6) [2].

From the point of view of epidemiological risk assessment, the multifactorial aetiology of MSD poses several challenges concerning the estimation of relative prevalence rates across occupational groups and potential implications for health inequalities among employed populations. In addition, epidemiological research has been focused on musculoskeletal disorders of the neck, lower back and upper limbs (e.g. shoulder, hand, arms, wrist), so that it is not clear to what extent the co-occurrence of known risk factors might differ between disorders of the upper and lower limbs (e.g. feet, ankles, knees). In this contribution these challenges are addressed by analysing cross-sectional data from the European Working Conditions Survey (EWCS) with the following research objectives: (1) to estimate the relative odds and prevalence rates of self-reported disorders of the upper limbs and/or shoulders and neck (upper body) and the lower limbs for major ISCO occupational groups, (2) to evaluate to what extent the associations between known risk factors differ for musculoskeletal disorders of the upper body and the lower limbs.

## Methods

### Samples and variables

The European Working Conditions Survey (EWCS) is a representative cross-sectional survey of workers (employees and self-employed) in the European Union conducted every five years [14]. The statistical population of the EWCS 2010 within each country represents all (non-institutionalised) persons aged 15 and over whose usual place of residence is in one of the countries included in the survey and who were employed the week that preceded the beginning of the interview. In general, the sampling scheme of the EWCS is a multistage stratified random sample. Strata are defined by NUTS regions level 2/3 or equivalent sampling units and, theoretically, all members of the population had a known non-zero inclusion probability [15]. In most countries the targeted sample was 1000 and the overall response rate for the survey was nearly 44%. The survey consists of a standardised questionnaire focusing on the working conditions and health status of workers across member states of the European Union and some other countries such as Norway, Switzerland or Turkey.

The descriptive and analytical analyses in this paper are restricted to the EU-15 countries for which data on physical working conditions is available (Austria, Belgium, Germany, Denmark, Spain, France, Finland, United Kingdom, Greece, Italy, Ireland, Luxembourg, The Netherlands, Portugal, and Sweden). Weighted descriptive statistics at the national and European level were estimated with EWCS waves 1995, 2000, 2005, and 2010 by using EU-15-specific sampling weights. Regression models were estimated with data from the EWCS 2010 as complete-case analyses. Only employed survey participants aged 18 to 65 years were included. The occupation of participants is operationalised by the International Standard Classification of Occupations 1988 (ISCO-88). Occupations are defined as a set of jobs whose main tasks and duties are characterised by a high degree of similarity. The ISCO-88 identifies the following 10 major occupational groups ISCO 1: managers, ISCO 2: professionals, ISCO 3: technicians and associate professionals, ISCO 4: clerical support workers, ISCO 5: service and sales workers, ISCO 6: skilled agricultural, forestry and fishery workers, ISCO 7: craft and related trade workers, ISCO 8: plant and machine operators, and assemblers, ISCO 9: elementary occupations, and ISCO 0: armed forces occupations [16]. In this paper ISCO 0 workers were excluded due to the fact that institutionalised persons are not covered appropriately in the EWCS waves.

### Regression models

Two generalised linear mixed models (GLMM) with logit link are estimated by using the Markov chain Monte Carlo Sampler in the framework of Bayesian inference [17–19]. These models are also known as mixed logistic regression or multilevel logistic models for dichotomous variables and were chosen in order to take into account the nested structure of the sampling scheme (i.e. countries and regions). The dependent variable of the first GLMM regression is a dichotomous variable corresponding to the EWCS survey question: “Over the last 12 months, did you suffer from muscular pains in shoulders, neck and/or upper limbs?”. The dependent variable in the second GLMM regression corresponds to the question: “Over the last 12 months, did you suffer from muscular pains in lower limbs?”. The independent variables include several physical and psychosocial risk factors that have been identified in the literature [2, 3, 6, 7, 20]. A description of the variables and summary statistics are reported in Tables 1 and 2. The original survey questions related to risk factors associated with MSD are reproduced in Table 1. Ordinal variables in the regression models were not dichotomised.

**Table 1:** **Working conditions dimensions covered by the EWCS across waves and considered in this paper**

Original question in EWCS	Abbreviation	Variable type
**Biomechanical/Physical factors**		
Does your main paid job involve tiring or painful positions?	Positions	Ordinal
Does your main paid job involve carrying or moving heavy loads?	Loads	Ordinal
Does your main paid job involve repetitive hand or arm movements?	Repetitive tasks	Ordinal
Does your job involve working at very high speed?	Speed	Ordinal
Are you exposed at work to high temperatures which make you perspire even when not working?	Temperature high	Ordinal
Are you exposed at work to low temperatures whether indoors or outdoors?	Temperature low	Ordinal
Are you exposed at work to vibrations from hand tools, machinery, etc.?	Vibrations	Ordinal
Does your main paid job involve standing?	Standing	Ordinal
**Psychosocial factors**		
Do you experience stress in your work?	Stress	Dichotomous
Do your colleagues help and support you?	Support	Dichotomous
Do you need further training to cope well with job duties?	Job skill demands	Polytomous
Does your main paid job involve handling angry clients, patients?	Contact with clients	Dichotomous
Are you able to choose or change your methods of work?	Job methods	Dichotomous
**Occupational health**		
Regarding the health and safety risks related to performance of your job, how well informed would	Informed	Ordinal
you say you are?

**Table 2:** **Weighted descriptive statistics**

Variable							Missing
							values
Age	42 (11.2)						0
Sex (%)	Male	Female					0
	51	49					
Upper body symptoms (%)	No	Yes					49
	55	45					
Lower limb symptoms (%)	No	Yes					52
	70	30					
Exposed to extreme physical working							
conditions Range: 1 (always) -7 (never)							
Positions	6 (1.9)						78
Loads	6 (1.7)						38
Repetitive tasks	4 (2.2)						55
Speed	5 (2)						117
Temperature/high	7 (1.4)						44
Temperature/low	7 (1.4)						58
Standing	4 (2.3)						45
Vibrations	7 (1.7)						37
Stress. Range: 1 (always) - 5 (never)	3 (1.1)						59
Shift work? (%)	No	Yes					135
	85	15					
Work hours per week	38 (11.6)						417
Support from colleagues (%)	(Almost) Always	Sometimes	Rarely/Never	Not applicable			123
	64	16	8	11			
Job skill demands (%)	Need training	Skills correspond	Not challenged				234
	11	58	31				
Contact with clients/customers? (%)	Yes	No					128
	70	30					
Able to change work methods? (%)	Yes	No					165
	70	30					
How well informed about work-related	2 (0.7)						438
risks? Range: 1 (well) - 4 (not at all)							
Travel time to work (minutes)	30 (36.1)						345
Occupation (ISCO 2008) (%)							180
	ISCO 1	9	ISCO 4	12	ISCO 7	11	
	ISCO 2	15	ISCO 5	18	ISCO 8	6	
	ISCO 3	16	ISCO 6	2	ISCO 9	10	
Company size. Number of employees (%)	Self employed	(1, 10]	(10,50]	(50,100]	(100,500]	+ 500	619
	11	28	28	10	14	8	
Job experience (years)	7 (9.5)						197
How often involved in sport, leasure	4 (1.6)						87
activities. Range: 1 (everyday) -6 (never)							
Country (%)							0
	BE	18	FR	14	AT	4	
	DK	5	IE	5	PT	4	
	DE	9	IT	7	FI	4	
	GR	5	LU	4	SE	4	
	ES	5	NL	4	UK	7	

Median, standard deviation in parentheses and proportions (%).

For the GLMM regressions, the Metropolis-Hastings and Gibbs sampler are used to update the models. The Markov Chain was iterated 13000 times with burn-in set at 3000. In order to take into account the clustered structure of the data, a nested random-effects structure is defined with regions nested within countries. An inverse-Wishart with parameters *V* = 1,*ν* = 0.002 is the prior distribution of the covariance matrix of the random-effects. The inverse-Wishart seemed appropriate since iterations series for each estimand did not suggest strong autocorrelations after 500 lags (range of autocorrelations: (−0.07, 0.09) and (−0.09, 0.09) respectively), and relative large effective sample sizes were obtained (ranges: (229, 391) and (173, 331) respectively). In addition, graphical inspection of the estimates in each iteration confirmed a stable mixing property of the chain after the 13000 iterations specified (graphics are available from the author). Predicted estimates of prevalence rates were obtained by marginalising over the random effects and residuals (*u*, *e*, respectively) and using the approximation

1 $$E_{u,e}(y)={\text{logit}}^{-1}\left(\frac{\mathrm{X\beta}}{\sqrt{1+{\left(16\sqrt{3}/15\pi \right)}^{2}\sigma^{2}}}\right)$$

where *E*(*y*) is the expected predicted value of the dependent variable (i.e. presence or absence of upper body and lower limbs complaints), *X* is the design matrix of covariates, *β* the vector of estimated parameters and *σ*^2^ the sum of the variance components [21]. In order to analyse the consistency of results and to test potential moderation effects of the occupational position of workers, the fully adjusted GLMM regressions are compared with the crude estimates obtained by simple logistic regression models ignoring random effects and excluding the occupational position of respondents. The GLMM regressions with logit link are estimated with the R-package MCMCglmm [17] and the crude estimates are calculated with the routines implemented in the function glm available for the programming language and statistical environment R. The analyses adhered to the STROBE guidelines for cross-sectional studies in epidemiology (see Additional file 1 for the detailed checklist of STROBE criteria) [22].

In order to evaluate the conditions of work across occupational categories and to compare the relative exposures of workers to adverse work environments, the so-called polar plots were used which allow a straightforward comparison of the relative weight of prevalences across groups. The polar plots are constructed for the EWCS waves by applying the following two-step procedure. First, the EU-15 weighted frequencies of the questions covering the physical conditions of work (see Table 1) are estimated for each wave and for each ISCO occupational category. Second, in order to consider only employees at high risk, the percentages of workers responding “All the time” and “Almost all the time” to the corresponding questions are added up for each wave *i* and each occupation *o*, say *ρ*_*i*,*o*_.

Each polar plot corresponds to a circle or radius 1 divided in segments representing each variable. The radius of the variable with the highest value of *ρ*_*i*,*o*_, say *ρ*_*i*,max_, is assigned the value 1 in wave *i*. All other values of *ρ*_*i*,*o*_ across ISCO categories are expressed as fractions of the highest value *ρ*_*i*,max_. In case that all occupations have the same score *ρ*_*i*,*o*_ in wave *i* (i.e. all workers being equally exposed), the circle would have radius 1 for all variables. For instance, the percentage of employees in the ISCO category 8 reporting in the EWCS 1995 being exposed “All the time” and “Almost all the time” to vibrations was highest (i.e. *ρ*_1995,max_=*ρ*_1995,ISCO 8_). Hence, for the ISCO category 8 the radius of the variable “Vibrations” is 1. Workers in other ISCO groups reported less frequently being exposed to vibrations so that all other radii have values lower than 1.

## Results

### Unequal burden of exposure

**Figure 1:**
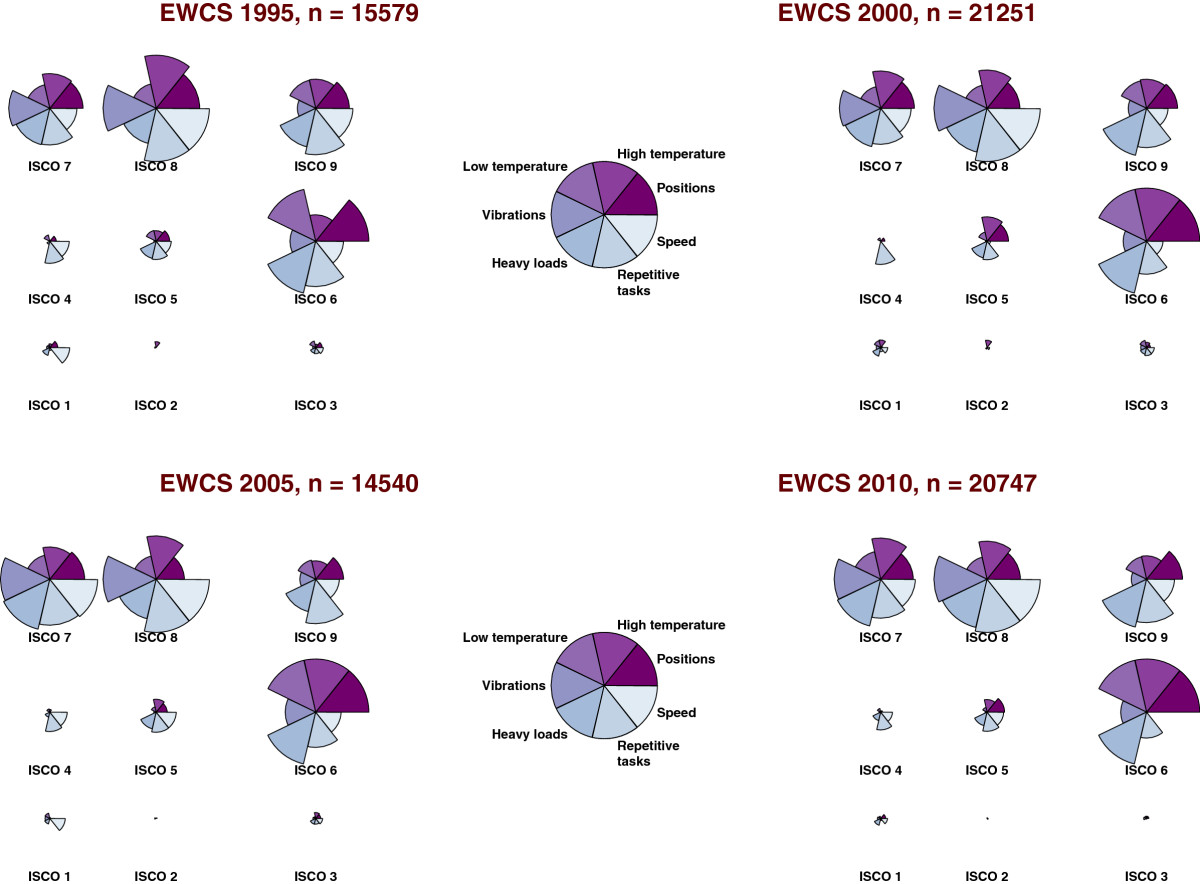
**Polar plots.** Relative proportion of workers reporting very frequent exposure to physical risk factors by ISCO-1988 occupational groups in the former EU-15 countries.

In Figure 1 the relative magnitude of the proportion of workers exposed to adverse physical working conditions across occupational categories is represented in the polar plots. A comparison of the estimates since EWCS 1995 reveals that the inequalities of exposure to physical risk factors remain not only consistent but also extremely concentrated in specific occupational groups. Agricultural workers (ISCO 6), craft workers (ISCO 7), operators (ISCO 8), and workers in elementary occupations (ISCO 9) report more frequently being exposed to adverse conditions in comparison with managers (ISCO 1), professionals (ISCO 2), technicians (ISCO 3), clerks (ISCO 4), and service workers (ISCO 5). Particularly, workers in ISCO categories 6, 7, and 8 report consistently very frequent exposure to tiring positions, extreme temperatures, carrying or moving heavy loads, working at very high speed and repetitive tasks. Service workers (ISCO 5) complain of being frequently exposed to tiring positions and carrying heavy loads. The percentage of managers, professionals, technicians and clerks reporting being frequently exposed to adverse physical working conditions across all waves is in comparison extremely small.

### Regression models

**Figure 2:**
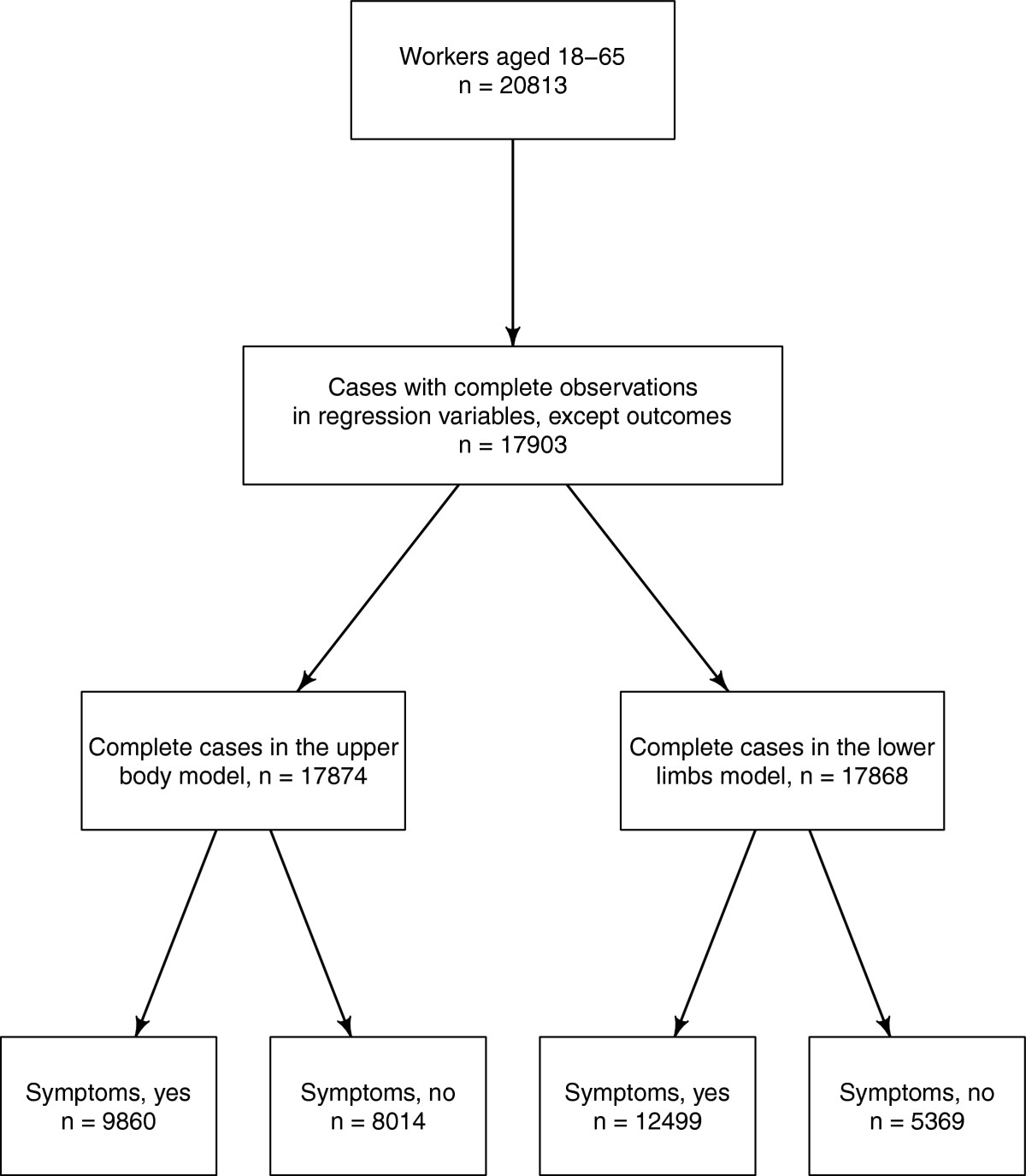
**Flow diagram of participants with complete information.** Selection of participants with complete information from the fifth European working conditions survey.

After row-wise deletion of observations with missing data the datasets underlying the regression analyses resulted in 17874 and 17868 complete cases, respectively, or about 84% of the original EWCS sample will all age groups (21201). The flow diagram of the complete-case datasets utilised in the regression models is reproduced in Figure 2. Descriptive statistics of the variables considered in the regression models are reported in Table 2. Prevalence of upper body and lower limbs complaints are large with upper body symptoms being more frequently reported than lower limbs complaints (45% vs. 30%). The median of the variables corresponding to exposure to tiring positions, carrying heavy loads, being exposed to very high or very low temperatures and vibrations is located at the right limit of the variable range (i.e. 7). These estimates suggest that most workers are not frequently exposed to those physical risk factors (see Table 2). About 27% of the EU-15 workforce is employed in occupations included in the ISCO groups 7, 8 and 9, whereas the majority of workers is employed in the professional, technical, clerical and services occupations (ISCO groups 2, 3, 4, and 5, respectively).

#### Musculoskeletal symptoms of the upper body

The results of the regression analyses estimating the probability of reporting musculoskeletal complaints of the upper body are summarised in Table 3. For an interpretation of the direction of the associations see the original survey questions in Table 1 and their corresponding scales reported in Table 2. Regarding physical working conditions, workers reporting very low exposure levels either to tiring positions, carrying heavy loads, performing repetitive hand movements or very low temperatures, are less likely to report symptoms. Among psychosocial risk factors, high stress levels, inadequate skills for the job tasks and customer/client contact are associated with a higher probability of musculoskeletal complaints. Workers who are not sufficiently informed about the health risks of their job, female employees and the self-employed are associated with higher prevalence rates of symptoms. A comparison between the crude estimates of the simple logistic regression and the GLMM estimates suggests that even after controlling for known physical and psychosocial risk factors, workers in ISCO categories 7 (craft workers), 8 (plant and machine operators) and 9 (elementary occupations) show an excess risk of reporting upper body pain.

**Table 3:** **Mixed logistic regression analyses modelling the probability of reporting musculoskeletal symptoms**

	Upper body and/or neck				Lower limbs			
Variables	Crude OR	95% CI	OR(G)	95% CI	Crude OR	95% CI	OR(G)	95% CI
Positions	0.80	[0.78, 0.81]	0.75	[0.73, 0.77]	0.83	[0.81, 0.84]	0.80	[0.78, 0.82]
Loads	0.92	[0.90, 0.95]	0.91	[0.89, 0.94]	0.92	[0.90, 0.94]	0.90	[0.87, 0.92]
Repetitive tasks	0.92	[0.90, 0.93]	0.90	[0.88, 0.92]	0.95	[0.94, 0.97]	0.96	[0.93, 0.98]
Speed	0.99	[0.97, 1.00]	1.00	[0.98, 1.03]	1.00	[0.98, 1.02]	1.00	[0.97, 1.02]
Temperature/high	0.98	[0.95, 1.00]	0.97	[0.94, 1.00]	0.96	[0.93, 0.98]	0.95	[0.92, 0.98]
Temperature/low	0.93	[0.90, 0.95]	0.92	[0.89, 0.95]	0.95	[0.92, 0.97]	0.94	[0.91, 0.97]
Vibrations	0.98	[0.95, 1.00]	0.99	[0.96, 1.03]	0.97	[0.94, 0.99]	0.98	[0.95, 1.02]
Standing	1.01	[1.00, 1.03]	1.01	[0.99, 1.03]	0.88	[0.86, 0.89]	0.87	[0.84, 0.89]
Stress	0.87	[0.85, 0.90]	0.80	[0.77, 0.83]	0.90	[0.87, 0.93]	0.83	[0.80, 0.86]
Shift work	1.08	[0.99, 1.19]	1.07	[0.95, 1.20]	1.09	[0.99, 1.21]	1.06	[0.94, 1.20]
Work hours per week	1.00	[1.00,1.00]	1.00	[1.00, 1.00]	1.00	[1.00, 1.00]	1.00	[1.00, 1.00]
Support from colleagues								
Always			Reference					
Sometimes	1.03	[0.94, 1.13]	1.02	[0.91, 1.15]	1.04	[0.95, 1.15]	1.03	[0.92, 1.16]
Rarely/never	0.93	[0.82, 1.05]	0.87	[0.74, 1.02]	0.99	[0.87, 1.13]	0.98	[0.84, 1.15]
No colleagues	0.82	[0.71, 0.95]	0.84	[0.71, 0.99]	0.91	[0.78, 1.06]	0.91	[0.75, 1.09]
Job skill demands								
Need training			Reference					
Match	0.88	[0.80, 0.98]	0.86	[0.76, 0.97]	1.00	[0.89, 1.12]	0.94	[0.81, 1.08]
Lower	0.88	[0.79, 0.99]	0.93	[0.81, 1.06]	1.03	[0.91, 1.16]	1.01	[0.87, 1.17]
No contact with clients	0.93	[0.87,1.00]	0.84	[0.77, 0.92]	0.95	[0.88,1.03]	0.88	[0.80, 0.97]
Unable to change work methods	0.91	[0.84, 0.98]	0.94	[0.86, 1.04]	0.96	[0.89, 1.04]	0.95	[0.85, 1.05]
Informed about risks	1.21	[1.15, 1.26]	1.19	[1.12, 1.25]	1.24	[1.19, 1.30]	1.24	[1.17, 1.31]
Travel time to work	1.00	[1.00, 1.00]	1.00	[1.00, 1.00]	1.00	[1.00, 1.00]	1.00	[1.00, 1.00]
Occupation								
ISCO 1			Reference					
ISCO 2			0.91	[0.76, 1.08]			0.81	[0.66, 0.98]
ISCO 3			1.08	[0.92, 1.27]			1.05	[0.86, 1.28]
ISCO 4			1.08	[0.90, 1.29]			1.19	[0.97, 1.46]
ISCO 5			1.03	[0.87, 1.24]			1.35	[1.13, 1.62]
ISCO 6			1.09	[0.76, 1.54]			1.29	[0.92, 1.80]
ISCO 7			1.30	[1.06, 1.58]			1.40	[1.16, 1.70]
ISCO 8			1.29	[1.04, 1.59]			1.41	[1.11, 1.79]
ISCO 9			1.37	[1.13, 1.66]			1.58	[1.28, 1.94]
Company size								
Self employed			Reference					
(1, 10]	0.78	[0.68, 0.90]	0.71	[0.59, 0.85]	0.78	[0.67, 0.91]	0.74	[0.62, 0.89]
(10, 50]	0.83	[0.71, 0.97]	0.74	[0.62, 0.90]	0.80	[0.68, 0.94]	0.77	[0.64, 0.93]
(50, 100]	0.84	[0.71, 1.00]	0.77	[0.62, 0.95]	0.68	[0.56, 0.81]	0.64	[0.52, 0.80]
(100, 500]	0.82	[0.70, 0.96]	0.77	[0.62, 0.94]	0.68	[0.57, 0.81]	0.66	[0.54, 0.82]
+ 500	0.87	[0.73, 1.05]	0.87	[0.70, 1.09]	0.78	[0.64, 0.95]	0.81	[0.64, 1.01]
Job experience	1.00	[1.00, 1.01]	1.00	[1.00, 1.01]	1.00	[1.00,1.00]	1.00	[1.00, 1.01]
Age	1.02	[1.02, 1.03]	1.03	[1.02, 1.03]	1.03	[1.03, 1.03]	1.04	[1.03, 1.04]
Female	1.53	[1.42, 1.64]	1.68	[1.53, 1.84]	1.26	[1.17, 1.37]	1.31	[1.19,1.44]
Sport/Leisure	0.92	[0.91, 0.94]	0.95	[0.92, 0.98]	0.99	[0.96, 1.01]	0.98	[0.96, 1.01]
n			17874				17868	
Deviance Information Criterion			21199				18715	
Country			0.28	[0.07, 0.57]			0.13	[0.03, 0.27]
NUTS region			0.25	[0.17, 0.32]			0.13	[0.07, 0.19]

Odds ratios (OR) and 95% CI from crude estimates (Crude OR) and estimates from the GLMM models (OR(G)).

#### Musculoskeletal symptoms of the lower limbs

The results of the regression analyses estimating the probability of reporting musculoskeletal complaints of the lower limbs are summarised in Table 3. For an interpretation of the direction of the associations see the original survey questions in Table 1 and their corresponding scales reported in Table 2. In a similar manner as for the upper body symptoms, very low exposure either to tiring positions, carrying heavy loads, performing repetitive hand or arm movements, very high or low temperatures or standing are associated with reduced chances of reporting symptoms. Workers dealing with customers and/or clients, females and workers lacking information about health risks of their job reported more frequently lower limbs symptoms. A comparison between the crude and fully adjusted models suggests also for lower limbs symptoms that there is still an occupational-specific excess risk even after adjustment. Service and sales workers (ISCO 5), craft workers (ISCO 7), machine operators (ISCO 8) and workers in elementary occupations (ISCO 9) reported more frequently lower limbs symptoms in comparison with managers (ISCO 1).

**Figure 3:**
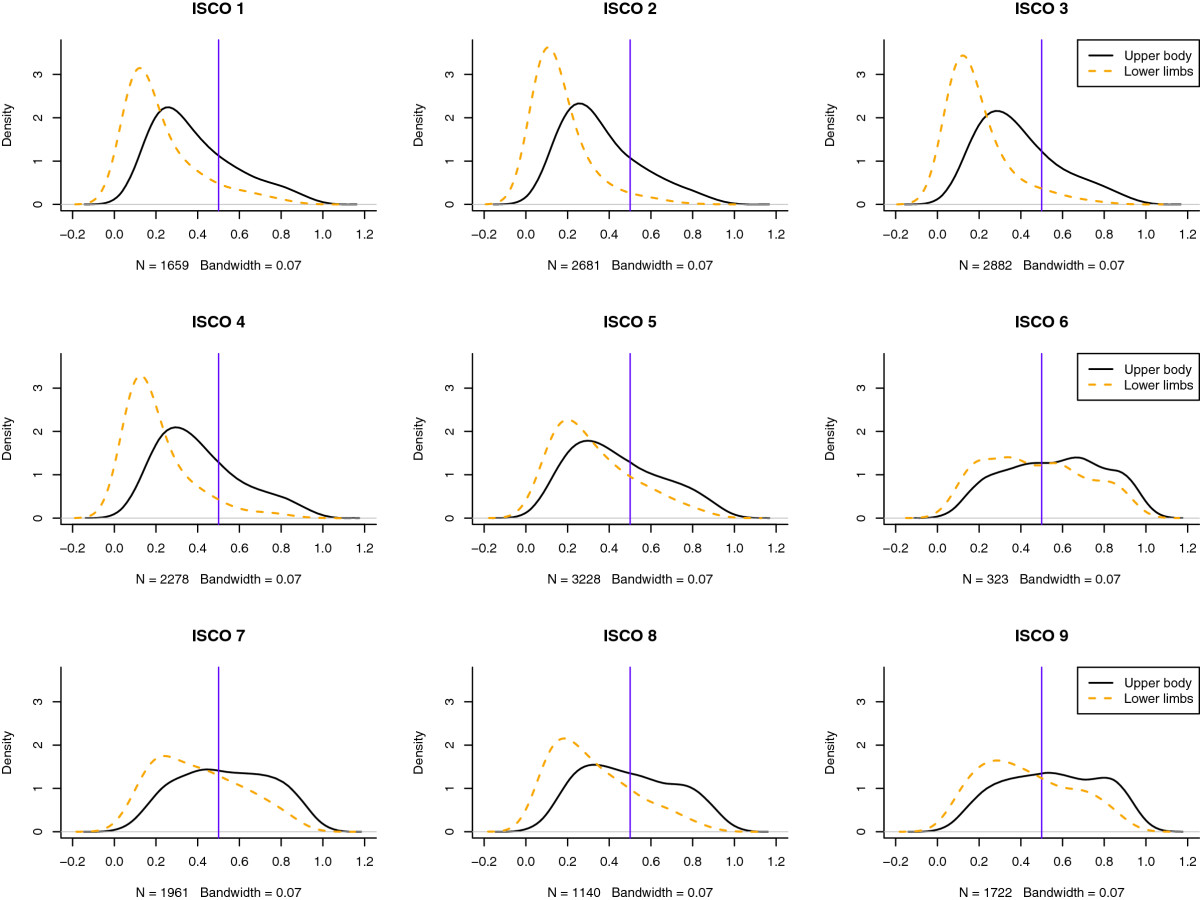
**Predicted distribution of musculoskeletal disorders by occupational class.** Kernel density estimates of the predicted distribution of musculoskeletal symptoms of the upper body (continuous line) and lower limbs (dotted line) by major ISCO occuptional groups. Plotted points lower than 0 or greater than 1 are not meaningful.

The kernel density estimates of the predicted probability of reporting musculoskeletal symptoms of the upper body and lower limbs across major occupational groups are depicted in Figure 3 (continuous and dotted lines, respectively). The curves in Figure 3 represent “smoothed” histograms only and were chosen in order to facilitate the graphical inspection of the marginal distribution of the GLMM regressions. Thus, a few plotted values slightly lower than 0 or greater than 1 are due to the kernel density estimation procedures and graphical representation constraints only. For comparison purposes, a vertical line in each panel is located at 0.5. Across occupational groups it can be seen that the probability of reporting symptoms increases substantially for workers in ISCO groups 5 to 9 in comparison to managers (ISCO 1), professionals (ISCO 2), technicians (ISCO 3) and clerical workers (ISCO 4). Within occupational groups, the mass of the distribution is concentrated on the left suggesting that the probability of reporting upper body symptoms is larger than the probability of reporting lower limbs symptoms. These differences can be compared with the raw and the predicted prevalence rates across occupational groups reported in Table 4. The raw prevalence rates were estimated from the EWCS 2010 and the predicted from Equation 1. Even though in general the raw and the predicted prevalence rates are larger among ISCO groups 5 to 9 than among ISCO groups 1 to 4, the raw and the predicted prevalence rates differ substantially for ISCO groups 1 to 4.

**Table 4:** **Raw and predicted prevalence rates of upper body and lower limbs symptoms by occupational group**

	Upper body symptoms (%)		Lower limbs symptoms (%)	
	Raw	Predicted	Raw	Predicted
ISCO 1	39	25	26	9
ISCO 2	39	22	20	4
ISCO 3	42	25	23	5
ISCO 4	42	28	24	6
ISCO 5	44	35	34	19
ISCO 6	55	59	50	46
ISCO 7	53	55	42	32
ISCO 8	50	47	34	19
ISCO 9	54	57	44	36

## Discussion

The objectives of this paper were (i) to estimate the relative odds and prevalence rates of self-reported musculoskeletal disorders of the upper body and the lower limbs for major ISCO occupational groups, (ii) to evaluate to what extent the associations between known risk factors differ for musculoskeltal disorders of the upper limbs and/or neck and the lower limbs. Concerning the first objective, an excess risk of reporting musculoskeketal disorders of the upper body was observed among craft workers (ISCO 7), machine operators (ISCO 8) and workers in elementary occupations (ISCO 9). Concerning musculoskeletal disorders of the lower limbs, service and sales workers (ISCO 5) and workers in ISCO groups 7, 8 and 9 reported symptoms more frequently. The statistically significant associations observed for each occupational group were consistent after adjusting for country-specific and regional variation and for some known physical, psychosocial and socio-demographic risk factors. These results reveal a social gradient of self-reported musculoskeletal complaints which is not being fully captured by the regression models considered here. On the contrary, even though skilled agricultural workers (ISCO 6) report very frequent exposure to known physical risk factors (see polar plots in Figure 1), the corresponding odds ratios reported in Table 3 suggest that the differences between workers in ISCO group 1 and ISCO group 6 are accounted for by the variables specified in the regression models.

Regarding the second objective, similar association patterns were observed for upper body and lower limbs symptoms. Major physical risk factors associated with both symptom types were very frequent exposure to tiring positions, carrying heavy loads and performing repetitive tasks. Standing appears to be an important risk factor for lower limbs symptoms only. High levels of self-reported stress, lack of information about occupational health risks, being self employed and female are also associated with increased risks of reporting both types of musculoskeletal disorders. Upper body and lower limbs symptoms are more frequently reported among ISCO groups 7, 8 and 9 in comparison with managers (ISCO 1).

The predicted prevalence rates reported in Figure 3 and Table 4 suggest that the fully adjusted prevalence rates may actually be lower for managers (ISCO 1), professsionals (ISCO 2), technicians (ISCO 3) and clerical workers (ISCO 4). By considering Equation 1 it can be seen that the prediction model takes into account the self-reported exposure levels and other relevant characteristics of survey participants. Since high exposure levels are much more frequent among workers of ISCO groups 6 to 9 (see polar plots in Figure 1), the predicted prevalence rates are actually not only reflecting the reporting of symptoms but also taking into account the inequalities of exposure across occupational groups.

The results of the previous analyses suggest that the unequal burden of exposure has not changed substantially across occupational groups since 1995, and that there is urgent need of delivering and evaluating the effects of specific interventions targeting workers at high risk of developing musculoskeletal disorders [2, 23–25].

### Limitations

This paper suffers from several limitations. (1) Causal relationships between risk factors and self-reported symptoms cannot be identified since cross-sectional data were analysed. For instance, the extensive systematic review of Mayer and colleagues based on longitudinal studies found strong evidence for the association between neck and/or shoulder complaints and vibration (OR between 1.6 and 2.5) [26]. In this paper, on the contrary, exposure to vibrations was not associated with upper body symptoms. This may be due to the fact that workers change jobs in order to avoid or reduce exposure to risk factors such as vibrations. (2) Exposure levels to physical risk factors in the EWCS are not assessed by biomechanical measurements at the workplace but rely on self-reported frequency scales. The instruments used to assess exposure to psychosocial risks are not psychometrically valid. These methodological deficiencies might bias the odds ratios and prevalence rates estimates, assess different constructs (e.g. coping instead of stress levels), and inflate or dilute real associations. (3) Even though all EWCS questionnaires in the different languages were based on the English version, there might be additional variance related systematically to translation and interpretation differences across countries and regions. However, this was taken into account to some extent by the nested structure of the regression models. (4) As stated in the Background section, the EWCS suffers from a large proportion of non-response (overall response rate about 44%). This might limit the generalisability of the results to the targeted statistical population in the selected countries. (5) The outcomes were not based on clinical diagnoses but on self-reported presence or absence of musculoskeletal pain in the last 12 months prior to the survey. Hence, there might be substantial misclassification of outcome status biasing the odds ratios and prevalence estimates.

## Conclusions

Exposure to known physical risk factors for musculoskeletal disorders is mainly concentrated on ISCO groups 6, 7, 8 and 9. This inequality of exposure has remained practically unaltered since 1995 in Europe (EU-15). At the same time, statistically significant differences of the prevalence of upper body and lower limbs complaints were observed across major ISCO occupational groups. In particular, service and sales workers (ISCO 5), craft workers (ISCO 7), machine operators (ISCO 8) and workers in elementary occupations (ISCO 9) reported more frequently musculoskeletal complaints. The results of the fully adjusted regression models suggest the existence of occupation-specific risk factors. Known physical and psychosocial risk factors seem to be relevant for both upper body and lower limbs musculoskeletal complaints.

## Electronic supplementary material

Additional file 1:Checklist of STROBE criteria.(PDF 9 KB)

Below are the links to the authors’ original submitted files for images.

Authors’ original file for figure 1

Authors’ original file for figure 2

Authors’ original file for figure 3

## Abbreviations

GLMM: Generalised linear mixed models
MSD: Musculoskeletal disorders
ISCO: International Standard Classification of Occupations
EWCS: European Working Conditions Survey
NUTS: Nomenclature of Units for Territorial Statistics
EU: European Union.
