# Supplementary material for: Upper body and lower limbs musculoskeletal symptoms and health inequalities in Europe: an analysis of cross-sectional data
Source: BMC Musculoskelet Disord. 2014 Aug 26;15:285. doi: 10.1186/1471-2474-15-285 (PMC4153890; doi:10.1186/1471-2474-15-285)
Supplement: Supplementary file 1 — Additional file 1:Checklist of STROBE criteria.(PDF 9 KB) [file 12891_2014_2230_MOESM1_ESM.pdf]

STROBE checklist for cross-sectional studies -
Upper body and lower limbs musculoskeletal symptoms and health inequalities in Europe. An analysis of cross-sectional data

	Item No	Recommendation	Manuscript
**Title and abstract**	1	(a) Indicate the study's design with a commonly used term in the title or the abstract	See title and abstract
		(b) Provide in the abstract an informative and balanced summary of what was done and what was found	See title and abstract
**Introduction**			
**Background/rationale**	2	Explain the scientific background and rationale for the investigation being reported	See Background section
**Objectives**	3	State specific objectives, including any prespecified hypotheses	See Background section
**Methods**			
**Study design**	4	Present key elements of study design early in the paper	See Methods section
**Setting**	5	Describe the setting, locations, and relevant dates, including periods of recruitment, exposure, follow-up, and data collection	Included. Data collection given by the EWCS methodology
**Participants**	6	(a) Give the eligibility criteria, and the sources and methods of selection of participants	Included. See also flow diagramme
**Variables**	7	Clearly define all outcomes, exposures, predictors, potential confounders, and effect modifiers. Give diagnostic criteria, if applicable	Included. Diagnostic criteria not applicable
**Data sources/ measurement**	8*	For each variable of interest, give sources of data and details of methods of assessment (measurement). Describe comparability of assessment methods if there is more than one group	All variables were collected by EWCS survey methodology
**Bias**	9	Describe any efforts to address potential sources of bias	Crude and fully adjusted models were estimated. Several known confounders were included. See Background section
**Study size**	10	Explain how the study size was arrived at	See flow diagramme
**Quantitative variables**	11	Explain how quantitative variables were handled in the analyses. If applicable, describe which groupings were chosen and why	See Table 1
**Statistical methods**	12	(a) Describe all statistical methods, including those used to control for confounding	See Methods section

STROBE checklist for cross-sectional studies -
Upper body and lower limbs musculoskeletal symptoms and health inequalities in Europe. An analysis of cross-sectional data

	Item No	Recommendation	Manuscript
		(b) Describe any methods used to examine subgroups and interactions	See Methods section
		(c) Explain how missing data were addressed	Only complete case observations were included. The number of missing data for the dependent variable is very low in comparison to sample size, see Table 2
		(d) If applicable, describe analytical methods taking account of sampling strategy	A nested random-effects structure by sampling units and countries was defined. See Methods section
		(e) Describe any sensitivity analyses	Crude and fully adjusted models were compared
**Results**			
**Participants**	13*	(a) Report numbers of individuals at each stage of study—eg numbers potentially eligible, examined for eligibility, confirmed eligible, included in the study, completing follow-up, and analysed	See flow diagramme, EWCS description and EWCS documentation referenced in the mansucript
		(b) Give reasons for non-participation at each stage	Not available
		(c) Consider use of a flow diagram	Included
**Descriptive data**	14*	(a) Give characteristics of study participants (eg demographic, clinical, social) and information on exposures and potential confounders	Included in Table 2
**Outcome data**		(b) Indicate number of participants with missing data for each variable of interest	Included in Table 2
**Main results**	15*	Report numbers of outcome events or summary measures	Included in Table 2
	16	(a) Give unadjusted estimates and, if applicable, confounder-adjusted estimates and their precision (eg, 95% confidence interval). Make clear which confounders were adjusted for and why they were included	Included in Table 3 (crude estimates)
		(b) Report category boundaries when continuous variables were categorized	Not applicable
**Other analyses**		(c) If relevant, consider translating estimates of relative risk into absolute risk for a meaningful time period	Not applicatble
**Key results**	17	Report other analyses done—eg analyses of subgroups and interactions, and sensitivity analyses	See Figure 3
**Limitations**			
	18	Summarise key results with reference to study objectives	See Discussion section
**Interpretation**	19	Discuss limitations of the study, taking into account sources of potential bias or	See Limitations in the Discussion section

STROBE checklist for cross-sectional studies -
Upper body and lower limbs musculoskeletal symptoms and health inequalities in Europe. An analysis of cross-sectional data

	Item No	Recommendation	Manuscript
		imprecision. Discuss both direction and magnitude of any potential bias	
**Generalisability**	20	Give a cautious overall interpretation of results considering objectives, limitations, multiplicity of analyses, results from similar studies, and other relevant evidence	See Discussion and Conclusion sections
**Other information**			
**Funding**	21	Discuss the generalisability (external validity) of the study results	See Discussion and Conclusion sections
	22	Give the source of funding and the role of the funders for the present study and, if applicable, for the original study on which the present article is based	Included in the Competing interests and Acknowledgements sections
