# Supplementary figures and images for: Upper body and lower limbs musculoskeletal symptoms and health inequalities in Europe: an analysis of cross-sectional data
Source: BMC Musculoskelet Disord. 2014 Aug 26;15:285. doi: 10.1186/1471-2474-15-285 (PMC4153890; doi:10.1186/1471-2474-15-285)

EWCS 1995, n = 15579

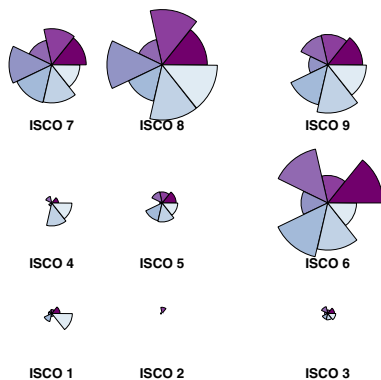

EWCS 2000, n = 21251

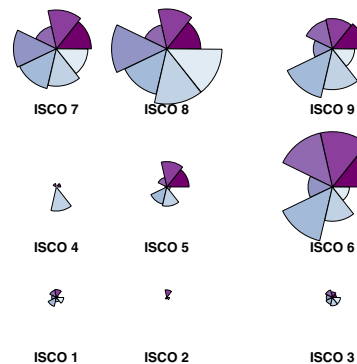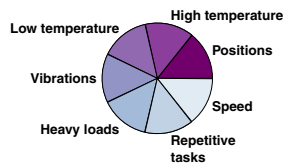

EWCS 2005, n = 14540

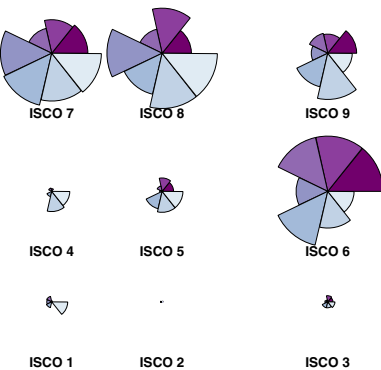

EWCS 2010, n = 20747

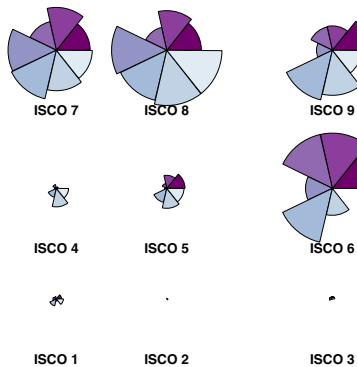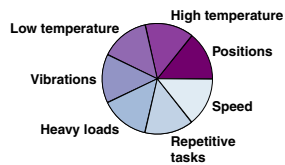

Supplement: Supplementary file 2 — Authors’ original file for figure 1 [file 12891_2014_2230_MOESM2_ESM.pdf]

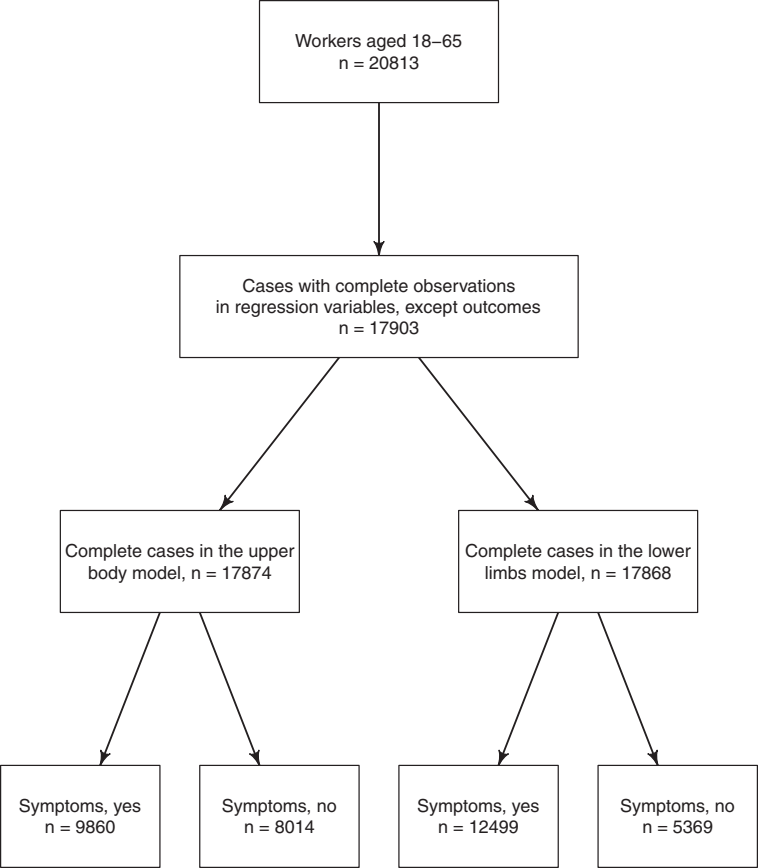

Supplement: Supplementary file 3 — Authors’ original file for figure 2 [file 12891_2014_2230_MOESM3_ESM.pdf]

ISCO 1

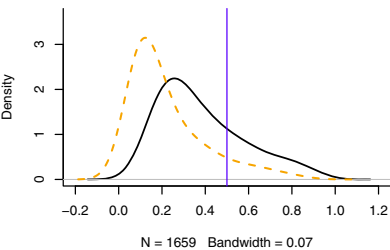

ISCO 2

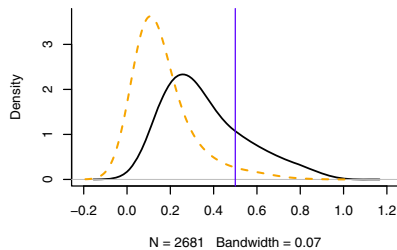

ISCO 3

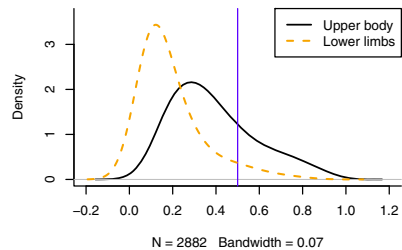

ISCO 4

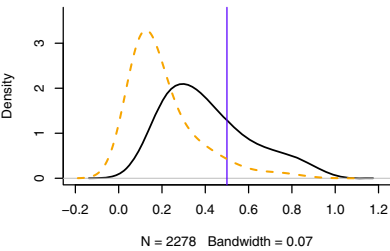

ISCO 5

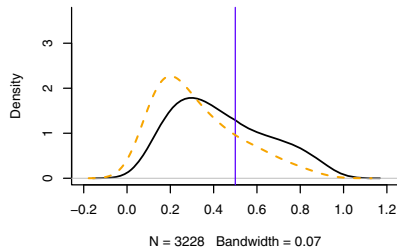

ISCO 6

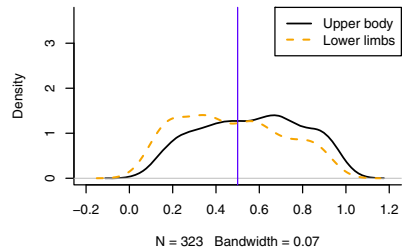

ISCO 7

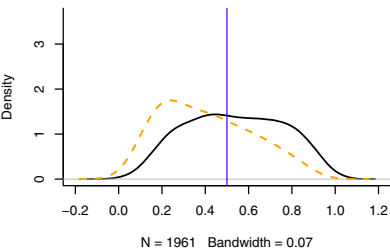

ISCO 8

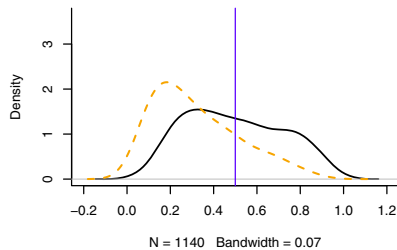

ISCO 9

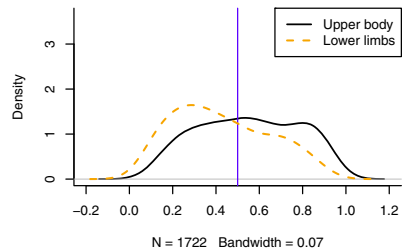

Supplement: Supplementary file 4 — Authors’ original file for figure 3 [file 12891_2014_2230_MOESM4_ESM.pdf]
